# Duration of chronic inflammation alters gene expression in muscle from untreated girls with juvenile dermatomyositis

**DOI:** 10.1186/1471-2172-9-43

**Published:** 2008-07-31

**Authors:** Yi-Wen Chen, Rongye Shi, Nicholas Geraci, Sheela Shrestha, Heather Gordish-Dressman, Lauren M Pachman

**Affiliations:** 1 Center for Genetic Medicine Research, Children's National Medical Center, Washington DC, USA; 2 Department of Pediatrics, George Washington University, Washington DC, USA; 3 The Cellular and Molecular Pathobiology program of The Children's Memorial Research Center, Northwestern University Feinberg School of Medicine, Chicago, IL, USA; 4 Department of Pediatrics, The Division of Rheumatology, The Children's Memorial Hospital, Northwestern University Feinberg School of Medicine, Chicago, IL, USA

## Abstract

**Background:**

To evaluate the impact of the duration of chronic inflammation on gene expression in skeletal muscle biopsies (MBx) from untreated children with juvenile dermatomyositis (JDM) and identify genes and biological processes associated with the disease progression, expression profiling data from 16 girls with active symptoms of JDM greater than or equal to 2 months were compared with 3 girls with active symptoms less than 2 months.

**Results:**

Seventy-nine genes were differentially expressed between the groups with long or short duration of untreated disease. Genes involved in immune responses and vasculature remodelling were expressed at a higher level in muscle biopsies from children with greater or equal to 2 months of symptoms, while genes involved in stress responses and protein turnover were expressed at a lower level. Among the 79 genes, expression of 9 genes showed a significant linear regression relationship with the duration of untreated disease. Five differentially expressed genes – HLA-DQA1, smooth muscle myosin heavy chain, clusterin, plexin D1 and tenomodulin – were verified by quantitative RT-PCR. The chronic inflammation of longer disease duration was also associated with increased DC-LAMP^+ ^and BDCA2^+ ^mature dendritic cells, identified by immunohistochemistry.

**Conclusion:**

We conclude that chronic inflammation alters the gene expression patterns in muscle of untreated children with JDM. Symptoms lasting greater or equal to 2 months were associated with dendritic cell maturation and anti-angiogenic vascular remodelling, directly contributing to disease pathophysiology.

## Background

Juvenile dermatomyositis, a systemic vasculopathy, is the most common of the inflammatory myopathies in children and is characterized by symmetrical proximal muscle weakness, and a pathognomonic rash, which includes a heliotrope discoloration about the eyes, dilated capillaries at the nailbeds and eyelid margins, and thickened erythema over the knuckles (Gottron's papules). The three other diagnostic criteria are: serum elevation of muscle derived enzymes, a muscle biopsy with specific histological features that include mononuclear cell infiltrates as well as perifasicular atrophy with evidence of an occlusive vasculopathy, and a positive electromyogram documenting inflammation [1].

There is little information describing the critical variables that influence the development and course of this often devastating illness. Over 3.2 cases/million children/year are diagnosed, with a 2.1 girl to 1 boy ratio [2]. At the time of their first symptom, rash or weakness, the mean age of the patient population is 6.7 years, while 25% of the children are age 4 or younger. It usually takes 4 months or more for the children to be diagnosed with JDM, when the muscle biopsy is obtained [3]. At diagnosis, the extent and severity of the skin and muscle inflammatory response can be assessed using validated disease activity scores (DAS) for skin and muscle involvement [2,4,5].

Children with JDM often have a family history of autoimmune disease. The frequencies of the HLA antigens DQA1*0501, DQA1*0301, and DRB3 have been reported to be higher in the JDM population than control populations suggesting a genetic contribution to this disorder, which may be additionally influenced by the TNF-α-308 allelic polymorphisms [6,7].

The precise stimulus initiating the inflammatory process is not known. However, there is evidence that newly diagnosed children with JDM have a history of infection, often respiratory or gastrointestinal in nature, within three months prior to the appearance of rash or muscle weakness [5]. In addition, gene expression profile data from MRI directed diagnostic biopsies of muscle from untreated children with active symptoms of JDM identified up-regulation of many type I interferon- (IFNα/β) inducible genes [8]. These findings support the interpretation that the inflammatory milieu in JDM is similar to that seen in anti-microbial responses. In that investigation we also found a marked down-regulation of genes associated with protein synthesis; both observations were subsequently confirmed in studies of muscle from adults with DM [9].

Untreated chronic inflammation in children with JDM is associated with the development of pathological calcifications [10]. The phenotype of the children who present in clinic early in their disease course differs from those diagnosed later, both with respect to height and weight, and specific JDM symptoms. Children diagnosed early in the disease course are much weaker than those who have a longer time to diagnosis [10]. However, the extent and severity of skin involvement appears to be stable over time [10].

With respect to diagnostic laboratory testing, serum levels of muscle enzymes, generally used to evaluate muscle inflammation, are more likely to be in the normal range when blood is collected 2–4.7 months after the child's first symptom (rash or weakness), making it more difficult to establish a diagnosis of definite JDM [10]. The purpose of the present study was to examine the impact of the duration of untreated chronic inflammation on gene expression in diagnostic muscle biopsies obtained from a large group of untreated girls with clinical symptoms of active JDM in order to identify genes and biological processes associated with disease progression in JDM.

## Results

### Demographics

All diagnostic biopsies studied were from untreated girls who had not been given any nonsteroidal or immunosuppressive therapy, and for whom a Disease Activity Score (DAS) was obtained at the time of biopsy (Table 1). The short disease duration group (mean 0.9 ± 0.3 months) was compared with the long duration group (mean 18 ± 27.9 months). Their ages at disease onset date were similar: 5.6 ± 1.0 years for the short duration group compared with 5.2 ± 2.7 years for long duration group. They were similar in disease activity scores. Total DAS for the short duration group was 11.7 ± 4.0 (mean DAS skin 5.0 ± 1.7, mean DAS muscle 6.7 ± 2.5) compared with the Total DAS for the long disease duration group of 12.0 ± 3.9 (mean DAS skin, 6.2 ± 1.4, mean DAS muscle 5.8 ± 3.1). Microarray analysis did not identify any gene significantly differentially expressed among different genotypes (TNF-α-308, DQA1*0301 and DQA1*0501). The second group of girls tested for confirmation of the gene expression changes by immunohistochemistry or RT-PCR, 5 patients each of long and short duration of untreated disease were also similar with respect to age at onset, race, and DAS scores (Table 2).

**Table 1:** Demographics of children with JDM in expression profiling study.

**Patient No.**	**Age at Onset**	**DAS Skin**	**DAS Weak**	**DAS Total**	**Age at MBx**	**Duration of Untreated Disease (Months)**	**DQA 0301**	**DQA 0501**	**TNF Type**
**Short Disease Duration**									
1	4.7	4.0	7.0	11.0	4.8	1.1	neg	pos	GA
2	6.8	7.0	9.0	16.0	6.8	1.1	neg	pos	GG
3	5.5	4.0	4.0	8.0	5.50	0.6	neg	pos	GA

Mean	5.6	5.0	6.7	11.7	5.7	0.9			
ST. Dev.	1.0	1.7	2.5	4.0	1.1	0.3			

**Long Disease Duration**									
4	1.0	6.0	5.5	11.5	3.5	30.6	neg	pos	GA
5	1.8	6.0	3.0	9.0	2.3	6.1	pos	neg	GG
6	8.3	9.0	10.0	19.0	8.5	2.1	neg	neg	GG
7	8.8	5.0	4.0	9.0	9.0	2.6	neg	pos	GA
8	3.9	6.0	9.0	15.0	5.2	16.1	neg	neg	GG
9	1.6	7.0	9.0	16.0	2.2	7.7	pos	neg	GG
10	7.2	8.0	6.0	14.0	7.4	2.2	pos	neg	GG
11	8.3	4.0	8.0	12.0	8.7	4.9	neg	pos	AA
12	6.0	7.0	3.0	10.0	7.7	20.6	pos	pos	GG
13	7.7	6.0	7.0	13.0	8.2	6.4	neg	pos	GG
14	2.5	3.0	1.0	4.0	3.6	13.6	neg	neg	GG
15	2.6	7.0	3.0	10.0	11.3	105.6	neg	neg	GA
16	6.1	7.0	10.0	17.0	6.6	6.6	neg	neg	GA
17	4.5	6.0	6.0	12.0	5.2	8.5	neg	pos	GG
18	5.1	6.0	8.0	14.0	10.3	63.5	neg	pos	AA
19	8.4	6.0	1.0	7.0	8.6	3.2	neg	pos	GG

Mean	5.1	6.2	5.8	12.0	6.7	18.8			
ST. Dev.	2.8	1.4	3.1	3.9	2.9	27.9			

DAS: disease activity score; MBx: muscle biopsy; DQA: Major histocompatibility complex, class II, DQ alpha-1; TNF type: Tumor necrosis factor -308 allele.

**Table 2:** Demographics of children with JDM in qRT-PCR validation.

**Patient No.**	**Age at Onset**	**DAS Skin**	**DAS Weak**	**DAS Total**	**Age at MBx**	**Duration of Untreated Disease (Months)**	**DQA 0301**	**DQA 0501**	**TNF Type**
**Short Disease Duration**									
1	6.8	5.0	9.0	14.0	6.9	1.2	neg	pos	GA
2	6.9	5.0	8.0	13.0	7.0	1.7	neg	pos	GG
3	6.1	7.0	9.0	16.0	6.3	1.8	neg	pos	GG
4	11.6	5.0	8.0	13.0	11.7	1.0	neg	pos	GG
5	8.9	5.0	7.0	12.0	9.0	1.5	neg	pos	GG

Mean	8.1	5.4	8.2	13.6	8.2	1.4			
ST. Dev.	2.2	0.9	0.8	1.5	2.2	0.4			

**Long Disease Duration**									
6	8.5	6.0	7.0	13.0	9.6	12.9	neg	neg	GG
7	5.6	8.0	8.0	16.0	5.9	4.0	neg	pos	GA
8	8.3	4.0	8.0	12.0	8.7	4.9	neg	pos	AA
9	7.7	6.0	7.0	13.0	8.2	6.4	neg	pos	GG
10	14.7	5.0	10.0	15.0	15.2	5.9	neg	pos	GG

Mean	9.0	5.8	8.0	13.8	9.5	6.8			
ST. Dev.	3.4	1.5	1.2	1.6	3.5	3.5			

DAS: disease activity score; MBx: muscle biopsy; DQA: Major histocompatibility complex, class II, DQ alpha-1; TNF type: Tumor necrosis factor -308 allele.

### Comparison of muscle biopsy gene profiles based on disease duration

As noted above, duration of disease, measured as more than 2 months of symptoms is a critical variable, influencing both clinical and laboratory data [10]. Since changes at the molecular level occur prior to phenotypic changes, we used 2 months (≥ 2 months compared with < 2 months) as the grouping cut-off in the present study. In order to control for gender effects, this study was limited to girls. We identified 79 genes represented by 85 Affymetrix probe sets differentially expressed in the muscle samples. All genes reached statistical significance (p < 0.05) after multiple testing correction with the false positive rate at 5%. In addition, in order to "bracket" the optimal cut-off time we compared data from patients with disease duration of 3 months or less with greater than 3 months, and also compared 4 months or less with greater than 4 months. There was no significant difference in expressed genes when the 4 month cut-off was used, and only 3 genes were differentially expressed when the 3 month cut-off was used.

**Figure 1:**
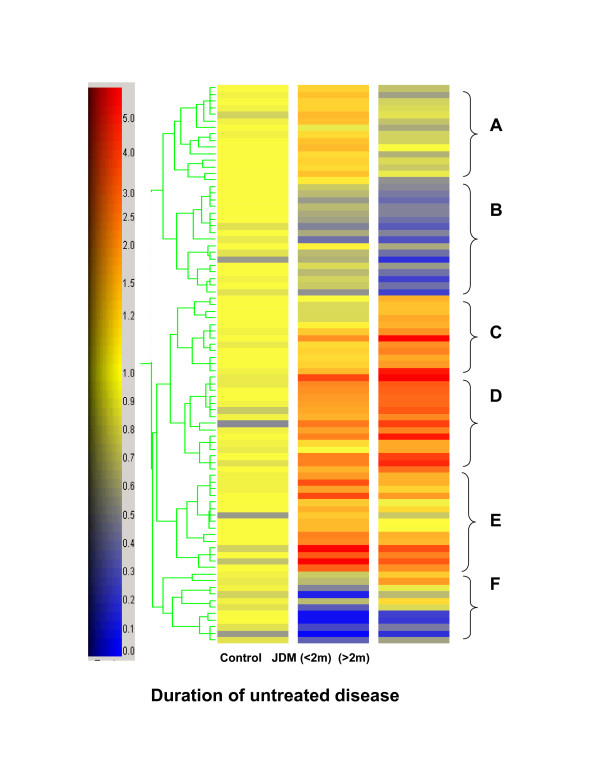
**Gene tree generated by hierarchical clustering based on gene expression patterns. **Genes up-regulated in girls with long (≥ 2 m) vs. short (< 2 m) duration of active disease were clustered into cluster C, D and F, while down-regulated genes were clustered into clusters A, B and E. The color codes represent the ratio between each of the JDM group compared with the age- and sex-matched control samples.

For an overview of the changes in relation to the baseline generated using the control samples from age-matched healthy girls undergoing cleft palate repair, we used hierarchical clustering analysis to visualize changes of gene expression patterns associated with the three groups (control, short and long active disease duration) (Figure 1). Six clusters were identified by visualizing the gene tree in Figure 1. Among the 79 genes, 44 genes were expressed at a lower level in the group with active disease greater than 2 months vs. shorter than 2 months (Figure 1, cluster A, B, and E), while 35 genes were expressed at a higher level in the group (Figure 1, cluster C, D, and F).

Cluster A represents genes up-regulated only in JDM patients with short duration of active disease. Cluster B represents genes significantly down-regulated only in JDM patients with long duration of active JDM. Many genes in these two clusters are involved in protein turn over and stress responses [see Additional file 1]. Most genes in cluster E are either encoding enzymes or proteins with unknown functions. These genes were highly up-regulated in patients with short duration of active disease but not in the other groups suggesting that they are activated at the acute phase of the disease.

For genes up-regulated in patients with longer duration of active disease (cluster C, D, and F), genes in cluster C and D were up-regulated in both groups when compared to healthy controls. However, the changes were significantly higher in the patients with long duration of active disease. Interestingly, most of the immune response genes identified in this study were grouped into these two clusters (Table 3). While genes in cluster F were expressed at higher level in the group with long duration, most of the genes in this cluster were down-regulated compared to the baseline (Table 4). In cluster F, most genes are associated with functions of vasculature remodelling. In this report, we will focus on genes in cluster C, D and F (Table 3 and 4), while the complete gene list of each cluster is reported in Additional file 1.

**Table 3:** Up-regulation of immune response genes in skeletal muscles of patients with active JDM longer than 2 months (Cluster C and D; genes are in order shown in figure 1)

**Affymetrix accession**	**p-value**	**Fold change (long/short)**	**Gene description**
**Cluster C**			
203981_s_at	6.2E-03	1.5	ATP-binding cassette, sub-family D (ALD), member 4
210105_s_at	1.5E-03	1.6	FYN oncogene related to SRC, FGR, YES
214430_at	1.4E-03	1.6	galactosidase, alpha
200602_at	6.5E-04	2.0	amyloid beta (A4) precursor protein
201103_x_at	5.7E-03	1.4	hypothetical protein LOC200030
209765_at	2.3E-03	1.6	a disintegrin and metalloproteinase domain 19
216510_x_at	9.6E-04	5.6	immunoglobulin heavy constant gamma 1
201069_at	3.9E-03	2.0	matrix metalloproteinase 2
203473_at	1.1E-04	2.2	solute carrier organic anion transporter family, member 2B1
203742_s_at	1.9E-03	1.5	thymine-DNA glycosylase
205917_at	6.5E-04	1.6	ZNF264
212671_s_at	2.5E-04	5.6	major histocompatibility complex, class II, DQ alpha 1/2
			
**Cluster D**			
218376_s_at	4.1E-03	2.5	NEDD9 interacting protein with calponin homology and LIM domains
209079_x_at	9.1E-04	1.6	protocadherin gamma subfamily
38671_at	5.9E-04	1.6	plexin D1
201279_s_at	2.0E-03	1.6	disabled homolog 2, mitogen-responsive phosphoprotein (Drosophila)
211066_x_at	6.5E-04	1.6	protocadherin gamma subfamily
215599_at	3.8E-04	2.1	SMA4
205717_x_at	1.8E-03	1.4	protocadherin gamma subfamily
217659_at	1.3E-03	1.6	KIAA0261
212607_at	1.1E-04	1.3	AKT3 (protein kinase B, gamma)
211748_x_at	4.0E-03	2.4	prostaglandin D2 synthase 21 kDa (brain)
215376_at	2.4E-04	1.4	CDNA FLJ12295 fis, clone MAMMA1001818
202259_s_at	1.1E-04	1.6	phosphonoformate immuno-associated protein 5
212187_x_at	2.1E-03	1.9	prostaglandin D2 synthase 21 kDa (brain)
206666_at	3.7E-04	2.5	granzyme K (serine protease, granzyme 3; tryptase II)
217947_at	5.9E-04	1.9	chemokine-like factor super family 6

**Table 4:** Genes involved in vasculature remodelling were down-regulated in patients with active JDM shorter than 2 months (Cluster F; genes are in order shown in figure 1).

**Affymetrix accession**	**p-value**	**Fold change ** **(long/short)**	**Gene description**
213290_at	4.2E-03	1.6	collagen, type VI, alpha 2
201141_at	2.1E-06	3.2	glycoprotein (transmembrane) nmb
221796_at	1.9E-03	2.0	cDNA clone IMAGE:452016
207695_s_at	1.9E-03	5.8	immunoglobulin superfamily, member 1
201369_s_at	6.7E-04	1.6	zinc finger protein 36, C3H type-like 2
222043_at	1.1E-04	2.9	Clusterin
201497_x_at	1.3E-03	7.9	myosin, heavy polypeptide 11, smooth muscle
207961_x_at	4.9E-04	6.4	myosin, heavy polypeptide 11, smooth muscle
204897_at	1.2E-03	1.9	prostaglandin E receptor 4
220065_at	6.5E-04	13.1	Tenomodulin
205573_s_at	5.0E-04	1.9	sorting nexin 7

In a previous profiling study, we identified a list of immune response genes differentially expressed in JDM patients, in which many of the genes were induced by type I interferon (IFNα/β) [8]. Although many probe sets on the U133A microarry are different from those on the Affymetrix FL microarray, the changes identified previously were verified using the new microarray. However we found no differential expression of those IFN inducible genes in the short and long disease duration group, suggesting the expression of these inflammatory response genes is independent of disease chronicity.

### Infiltration of mature dendritic cells in the patients with longer duration of active symptoms

To interpret the changes of genes involved in immune responses in cluster C and D (Table 3), we first searched for genes that were highly expressed in specific immune cell types. Although most of the genes were not specifically expressed by a single cell type, HLA-DR and glycoprotein nmb have been shown to be highly expressed in dendritic cells compared to B cells, monocytes and T cells [11]. This pattern of up-regulation of the genes suggested that there might be a higher number of dendritic cells in the muscle of children with a longer duration of active disease.

Using qRT-PCR, we first verified the expression changes HLA-DQA1 which could be either directly involved in antigen presentation or a surrogate marker of an active site. Because supply of diagnostic muscle biopsy tissue was limited, we tested a different group of 5 girls with short duration of disease, less than 2 months and a different group of 3 girls with disease duration greater or equal to 2 months, plus 2 girls from the original long disease duration group, which had been gene profiled in this study (Table 2).

**Figure 2:**
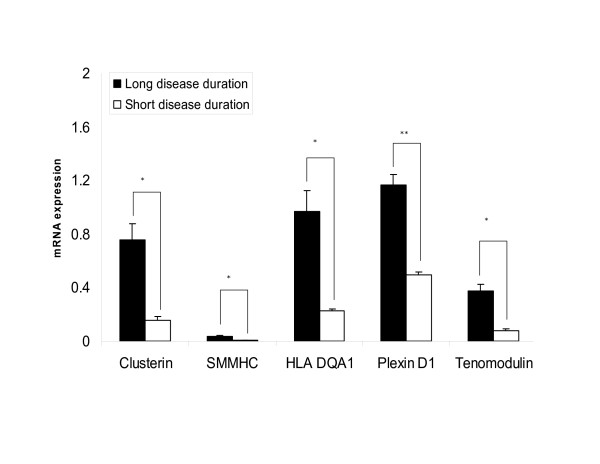
Five differentially expressed genes, HLA-DQA1, smooth muscle myosin heavy chain (SMMHC), clusterin, plexin D1, and tenomodulin were verified by quantitative RT-PCR, and their level of expression compared in diagnostic muscle biopsies from 5 girls with untreated symptoms of JDM for a short disease duration < 2 months (open bars) and 5 girls with untreated symptoms of JDM of ≥ 2 months duration (black bars). * p < 0.05, **p < 0.005.

**Figure 3:**
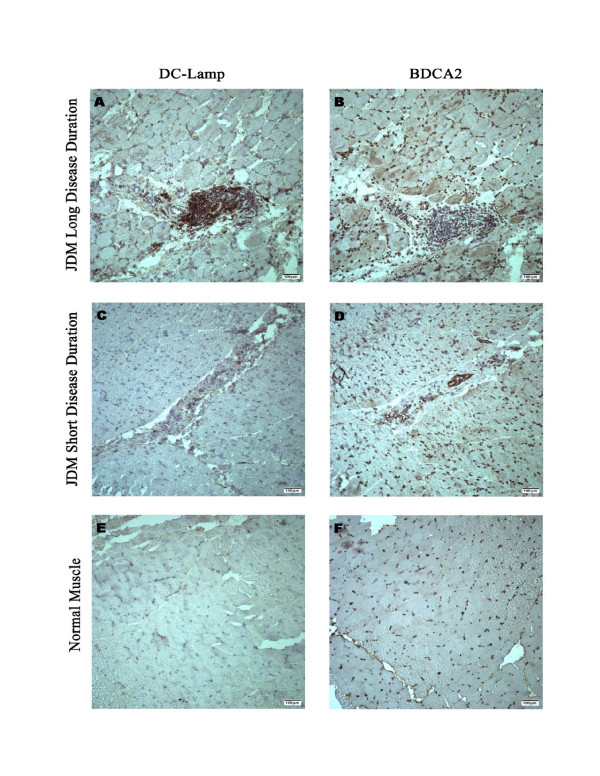
**Comparisons of dendritic cells in skeletal muscle biopsies from JDM, long and short disease duration, and normal patients.** DC-LAMP is a membrane bound protein produced by mature activated dendritic cells, while BDCA2 is an antigen produced by immature plasmacytoid dendritic cells (both markers are stained dark brown). Long disease duration JDM patients displayed a greater overall presence of mature dendritic cells (A) compared to short disease duration JDM patients (C). Greater concentrations of mature dendritic cells were found in perivascular and perifasicular regions compared with the endomysium. Many mature dendritic cells co-expressed plasmacytoid markers (A, B). No substantiated differences were found in the distribution of the BDCA2 positive plasmacytoid dendritic cells in JDM of either long or short disease duration (B, D). Normal pediatric muscle displayed an absence of mature dendritic cells with presence of BDCA2 positive cells (E). Images were taken at 10× on a Leica Upright Light Microscope. Scale bars represent 10 μm.

The expression level of HLA-DQA1 was 4.28 fold (p < 0.05) up-regulated in patients with longer duration of active disease, which verified our array data (5.6 fold, p < 0.005) (Figure 2). We then performed immunohistochemistry, using an antibody directed against a dendritic cell maturation marker, DC-LAMP to localize the cells in the muscle tissues (Figure 3). Long disease duration JDM patients displayed a greater overall presence of mature dendritic cells (Figure 3A) compared to short disease duration JDM patients (3C). Greater concentrations of mature dendritic cells were found in perivascular regions compared with the endomesium, In addition, many mature dendritic cells were BDCA2 positive suggesting a plasmacytoid origin. No significant differences were found in the distribution of the BDCA2 positive plasmacytoid dendritic cells in JDM of either long or short disease duration (B, D). Muscle from healthy children displayed an absence of mature dendritic cells (E), but a similar display of BDCA2 positive cells (F) compared with JDM.

### Vasculature remodelling in JDM patients

Based on the array data, genes involved in vasculature remodelling, including the structural gene, SMMHC (two probe sets, 7.9 and 6.4 folds, p < 0.005), and regulatory factors, tenomodulin (13.1 fold, p < 0.001), prostaglandin E receptor 4 (1.9 fold, p < 0.005), plexin D1 (1.6 fold, p < 0.001), Akt-3 (1.3 fold, p < 0.001), clusterin (2.9 fold, p < 0.001), and LPGDS (two probe sets, 2.4 and 1.9 folds, p < 0.005) [12-19], were significantly differentially expressed between patients with long and short duration of active disease, suggesting the involvement of vasculature remodelling during this period of time (Table 3 and 4). In addition, matrix metalloproteinase 2 (2.0 fold, p < 0.005) and collagen VIα2 (1.6 fold, p < 0.005) were also up-regulated indicating extracellular matrix remodelling, which is part of angiogenesis [20,21]. Interestingly, most of these genes belonged to cluster F, in which genes were down-regulated more in patients with short duration of active disease when comparing to the baseline (Figure 1, cluster F), suggesting that the expression changes of these specific genes were preferentially misregulated in the earlier stage of the inflammatory disease process.

Using qRT-PCR, we confirmed the higher expression of 4 genes related to vascular remodelling in muscle biopsies of girls with longer disease duration compared with disease of short duration. The expression level of smooth muscle heavy chain myosin was elevated 5.9 fold (p < 0.05), clusterin was 4.74 fold increased (p < 0.05); the level of plexin D1 was increased in the long duration group by 2.35 fold (p < 0.005), and tenomodulin was increased by 4.6 fold, (p < 0.05) (Figure 2).

A regression analysis was performed of 79 genes and 9 genes (10 probe sets) were identified that had a statistically significant linear relationship between the level of gene expression and duration of active disease (Table 5). Both probe sets representing SMMHC showed linear relationships, suggesting that the gene expression changes are dependent on the duration of the chronic inflammation of active disease.

**Table 5:** Nine genes (10 probe sets, SMMCH are represented by two probe sets) for which the expression level correlated with the duration of untreated duration of JDM

**Affymetrix accession**	**Gene description**	**r square**	**p-value**	**Cluster**
207961_x_at	myosin, heavy polypeptide 11, smooth muscle	0.47	0.002	F
201369_s_at	zinc finger protein 36, C3H type-like 2	0.40	0.005	F
203742_s_at	thymine-DNA glycosylase	0.39	0.006	C
214430_at	galactosidase, alpha	0.34	0.011	C
201497_x_at	myosin, heavy polypeptide 11, smooth muscle	0.33	0.013	F
204807_at	transmembrane protein 5	0.28	0.025	A
214437_s_at	serine hydroxymethyltransferase 2 (mitochondrial)	0.27	0.027	E
215376_at	CDNA FLJ12295 fis, clone MAMMA1001818	0.27	0.027	D
202854_at	hypoxanthine phosphoribosyltransferase 1 (Lesch-Nyhan syndrome)	0.25	0.034	E
221931_s_at	SEH1-like (S. cerevisiae)	0.23	0.042	A

## Discussion

### Impact of duration of untreated inflammation on clinical and laboratory findings in children with JDM

Previous studies have confirmed the observation that prolonged duration of untreated symptoms in children with JDM are highly associated with the development of pathologic calcifications, one of the most debilitating complications of JDM [22,23]. Untreated children with a longer duration of chronic symptoms had a higher frequency of loss of nailfold capillary end row loops [24], which persisted after 36 months of therapy [25]. The chronic inflammation associated with nailfold capillary loss was accompanied by impaired absorption of orally administered corticosteroids [26]. In addition, chronic untreated inflammation at diagnosis of JDM was associated with severe bone loss and an abnormal osteoprotegerin: RANKL ratio [10]. A study of 166 previously untreated children with JDM enrolled in a large national NIAMS JDM Research Registry showed that the clinical presentation of the children with shorter duration of active JDM was quite different from those who came to diagnosis later in their disease course. Children diagnosed early in the disease course were more likely to be weaker than later on, and muscle enzymes were more likely to be in the normal range 2–4.7 months after the child's first symptom (rash or weakness) [10] which may be associated with differential utilization of the TRAIL apoptotic pathway [10,27]. In this study we used a 2 month cut-off and identified 79 genes differentially expressed in patients with short (less than 2 months) and long (greater than 2 months or equal) duration of active disease by expression profiling. Identifying these changes provide insight concerning the observed differences in clinical phenotypes at the molecular level over time.

### Down regulation of genes involved in protein turnover and metabolism

Down-regulation of protein turnover and stress response genes in skeletal muscles has been associated with muscular dystrophies and conditions associated with muscle atrophy [28,29]. In this study, genes involved in both protein turnover and stress responses were down-regulated significantly in patients with longer duration of active disease [see Additional file 1]. The genes encoding heat shock proteins (eg. heat shock 70 kDa protein 9B and heat shock 40) and genes involved in proteasome functions (eg. proteasome regulatory particle subunit p44S10, ubiquitination factor E4B, located in cluster A) showed slight upregulation in the short duration group compared to controls, but approached the baseline in the long duration group, reflecting the acute phase of the disease. In contrast, genes involved in protein synthesis (eg. eukaryotic translation initiation factor 2, mitochondrial ribosomal protein S10 and L15) were mildly downregulated in the short duration group and achieve significant down-regulation in the long duration group, suggesting continuous suppression of protein synthesis commonly associated with muscle atrophy. The changes at the molecular level support the observation of ongoing muscle loss in untreated patients with JDM. Gene involved in ER stress response have been previously reported to be differentially expressed in adults with inflammatory myopathies [30]. In our study, we did not observe significant differences between the groups, based on the duration of untreated disease.

### Dendritic cell infiltration

The presence of dendritic cells in the muscle of patients with dermatomyositis was previously reported [31,32]. Recent investigation presented data showing that the localization and maturation of resident plasmacytoid dendritic cells *in situ *in the perivascular areas was essential to the initiation and perpetuation of muscle inflammation in juvenile DM [31]. The present study confirms that the typical pattern of dendritic cell infiltration (with heavy infiltration surrounding the blood vessels) in JDM is a progressive process associated with persistent inflammation without focal infiltration in muscle of patients with short duration of active disease. Many DC-LAMP positive dendritic cells were positive for BDCA2, again confirming that they were of plasmacytoid dendritic cell lineage as recently reported [31]. Dendritic cells are well characterized for efficient antigen presentation to T cells and are speculated to affect both tolerance and T cell activation [33]. Models of autoimmunity through activated dendritic cells in rheumatoid arthritis and in systemic lupus erythematosus (SLE) associated with a Type 1 IFN-α/β initiated response have been proposed by other investigators [34-36]. In addition, increased expression of IFNα/β induced genes have been identified in peripheral blood of children with SLE, systemic onset juvenile rheumatoid arthritis, and in JDM [37-39]. In this study, we found large number of dendritic cells in perivascular areas with dense mononuclear infiltrates in biopsies from children with longer duration of active disease, which suggested that dendritic cells might be actively involved in the progression of the disease by modulating T cells function in the muscle vasculature.

### Vasculature remodelling

Vascular smooth muscle cells (SMCs) are highly plastic capable of profound alterations in phenotype in response to changes in local environmental cues [40]. Vascular injury initiates a transition in the phenotype of vascular SMCs whereby "contractile" (differentiated) SMCs are capable of undergoing transient modification to a highly "synthetic" (dedifferentiated) state. These synthetic vascular SMCs are migratory, highly proliferative, and play a critical role in repair of the vascular injury. Upon resolution of the injury, SMCs reacquire their contractile phenotype and associated markers, which include smooth muscle isoforms of contractile apparatus proteins such as SMMHC [40]. In this study, we found clusterin, tenomodulin and prostaglandin E receptor 4 were down-regulated in JDM patients with short disease duration. In contrast, for children with longer duration of active disease, expression of these genes approached the baseline, and additional genes involved in vasculature remodelling were up-regulated, (plexin D1, Akt-3, LPGDS). These genes are associated with contractile (differentiated) vascular SMCs. These data suggest that early in the course of JDM, a relatively high proportion of vascular SMCs at the site of inflammation are in a synthetic (dedifferentiated or undifferentiated) state; conversely, as time passes and the inflammation becomes chronic, vascular SMCs further differentiate. The linear regression relationship between the duration of active disease and the SMMHC expression suggests that the vasculature remodelling in the muscles is a progressive process associated with disease chronicity and the duration of active disease. This is consistent with the documentation of evidence of cardiovascular compromise in older patients who had JDM in childhood [41]. Among the genes involved in vasculature remodelling, plexin D1 and prostaglandin E receptor 4, are two potent angiogenic factors [12,19]. Plexin D1 is expressed in vascular endothelial cells of developing blood vessels. Signalling between semaphorin 3E and its receptor plexin D1 controls endothelial cell positioning and the patterning of the vasculature during embryonic development, and can negatively regulate angiogenesis as well as activate T cells [42]. Because the hallmark of JDM is occlusion and obliteration of capillaries and arterioles, it is not surprising that expression of tenomodulin is increased, for it is overexpressed in hypovascular connective tissue. The up-regulation of these genes might underlie the molecular mechanisms of the vasculature loss and remodelling we observed in the patients' muscles.

In summary, the findings from this study support the clinical observation that identification of the duration of the inflammatory process is a critical component in children with JDM, influencing both the gene expression and the pathophysiology of the immune responses in children with active symptoms. Recognition of this variable, which identifies the chronicity of the process, is important in dissecting out factors involved in immune progression and response.

## Conclusion

We conclude that the duration of the chronic inflammatory process in untreated JDM alters mRNA expression patterns, including both dendritic cell maturation and vascular remodelling with increased expression of anti-angiogenic factors. We propose that the duration of the inflammatory process must be considered when interpreting gene profiling data as well as clinical and laboratory findings in children with JDM in order to gain insight into potential modes of intervention. We speculate that interventions that diminish the antiangiogenic remodelling present in children with JDM who have a longer duration of untreated disease may be of benefit.

## Methods

### Patient population

Age appropriate informed consent was obtained from a total of 31 girls with definite/probable JDM (IRB# 2002-11762) and the 4 healthy age and sex-matched controls (IRB# 2001-11715) who were enrolled in this study. Ethical approval for this study was obtained from the Children's Memorial Hospital Institutional Review Board, and all procedures were carried out in accordance with the Helsinki Declaration.

All the girls with JDM were negative for myositis specific or associated antibodies or for antibodies indicating overlap syndromes at the time of biopsy, and, as part of their diagnostic evaluation, had an MRI directed muscle biopsy. In the first step, 23 muscle biopsies from partially treated (n = 4) patients and untreated (n = 19) JDM patients and 4 control biopsies were expression profiled and used for gene filtering. In the second step, only untreated JDM patients at the time of muscle biopsy (n = 19, 3 short and 16 long duration) were analyzed statistically. In the third step, specific genes identified by the expression profiles were confirmed by testing additional samples from 8 untreated children with JDM. There was enough muscle biopsy material from 2 of the rare group of children with disease duration less than 2 months to be used for qRT-PCR enabling comparison of 5 patients each with long and short disease duration, as well as immunohistochemical studies.

The date of recognition of the first symptom (rash or weakness) was defined as the "disease onset date". The "duration of disease" at the time of the muscle biopsy was defined as the time from disease onset to the date of muscle biopsy. We have reported the results from profile analyses performed using only samples from untreated girls with JDM; their demographics are presented in Table 1.

Confirmation of the gene profiles by q-RT PCR utilized a separate set of 5 muscle biopsies from girls with a short duration of disease (1.4 ± 0.4 SD months), matched with 5 muscle biopsies' from girls with a long duration of untreated symptoms (6.8 ± 3.5 SD months); 2 children tested were also part of the gene profile long duration group. The girls were matched for age (8.2 ± 2.2 years compared with 9.5 ± 3.5 years), as well as disease activity, with respect to DAS skin (5.4 ± 0.9, 5.8 ± 1.5 respectively) and DAS muscle (8.2 ± 0.8, 8.0 ± 1.2 respectively). Details are presented in table 2.

The four control muscle from non-inflammatory female donors were biopsies, obtained with informed consent, from thoracic muscle from girls undergoing plastic surgery for repair of cleft palate, which do not appear to have evidence of the genetic dysregulation observed in JDM [43]. Their age range was 8–10.3; their mean age was 9.5 years.

### Disease Activity Scores

Overall measure of severity of the JDM disease activity was assessed at the time of the diagnostic muscle biopsy using the total disease activity score (DAS), a 20 point scale which has two sub-scales, which reflect skin involvement (ranging from 0–9) and muscle inflammation (ranging from 0–11) [4]. The skin component (DAS skin) is based on extent and severity of rash, the presence of telangiectasia (nailfold, palate, eyelids) and Gottron's papules [4] The muscle component (DAS muscle) includes measures of muscle function and the extent of weakness in eight manoeuvres as evaluated by a single physician (LMP) on routine diagnostic physical examination. Both sub scores have been validated for inter-rater reliability.

### Muscle biopsy samples

A diagnostic muscle biopsy, frequently the vastus lateralis, was obtained from the area of inflammation as defined by an MRI, using a T-2 weighted image with fat suppression. The sample was divided so that one portion was saved for immunohistochemical studies, while the other portion of the sample was used for gene expression profile studies and gene confirmation. Both parts of the muscle biopsy samples were snap frozen and stored in liquid nitrogen (-180°C).

### Determination of DQA1*0501 and DQA1*0301

At the diagnostic visit, immediately prior to the muscle biopsy, peripheral blood mononuclear cells were obtained by Ficoll-Hypaque separation, and frozen in liquid nitrogen at -180°C.

Genomic DNA was extracted from whole blood or frozen lymphocytes using the Puregene DNA Purification Kit (Gentra Systems, Minneapolis, MN). DNA from JDM patients was genotyped for HLA-DQA1*0301 and HLA-DQA1*0501 alleles using PCR amplification with sequence-specific primers as previously reported [44,45]. Aliquots of DNA (2 μl) were amplified using *Taq*Bead™ Hot Start Polymerase wax beads (1.25 U/bead; Promega Corporation, Madison, WI) in a reaction volume of 50 μl that contained 5× Green GoTaq™ Reaction Buffer (Promega Corporation, Madison, WI), dNTPs (0.2 mM each), and 0.4 μM allele specific primers for HLA-DQA1*0301 or HLA-DQA*0501.

The primers for HLA-DQA1*0301 were: 5'-TTCACTCGTCAGCTGACCAT-3' (Forward) and 5'-CAAATTGCGGGTCAAATCTTCT-3' (Reverse), which amplify a 183 bp product. The primers for HLA-DQA*0501 were: 5'-ACGGTCCCTCTGGCCAGTA-3' (Forward) and 5'-AGTTGGAGCGTTTAATCAGAC-3' (Reverse), which amplify a 186 bp product.

Denaturation was performed in each PCR at 94°C for 45 seconds, annealing at 62°C for 45, and extension at 72°C for 2 min (for the final step, extension was for 7 min). Absence or presence of PCR products was visualized by agarose gel electrophoresis.

### Determination of the TNF-α-308 polymorphism

The TNF-α-308 polymorphism consists of a single base pair G to A substitution. PCR was used to amplify a 107 bp fragment that incorporated the polymorphic site into an NcoI restriction site as previously described [23,45] distinguishes AA from AG from GG.

### Expression profiles of JDM patient muscle biopsies

Total RNA was isolated from each biopsy, processed for production of biotinylated cRNA and hybridization to microarrays, as we have previously described [28]. Each sample was then hybridized to Affymetrix U133A microarrays containing approximately 14,500 well-characterized transcripts. Standard operating procedure and quality control was done as previously described [46]. Muscle samples from 23 female JDM patients and 4 healthy age- and sex-matched controls were initially profiled.

Generation of hybridization signals (probe set algorithms) of the microarrays was done using Affymetrix MAS (Version 5.0) (Affymetrix, CA), and dCHIP [47]. After the absolute analysis, the gene expression levels were imported into GeneSpring software. The JDM samples were normalized to the mean of the profiles of age- and sex-matched control samples. Data filtering was done by retaining only those probe sets that showed at least two MAS5.0 "present calls" across all profiles. This resulted in retention of 67% of probe sets for the U133A microarrays.

Welch t-test was used to calculate the probabilities of significant gene expression changes between samples with shorter (< 2 months) and longer (≥ 2 months) disease duration. To reduce false positives, correction for multiple testing was done using Benjamini and Hochberg false discovery rate (5%) [48,49]. In addition, we used all treated and untreated patients to first generate a gene list with genes that showed statistically significant changes to reduce number of genes for multiple testing. We then used only the profiles of untreated patients and performed t-tests on the filtered genes with multiple testing corrections to minimize the false positives. To visualize transcripts showing coordinate regulation as a function of active disease duration, genes sharing temporal patterns were identified by hierarchical clustering using GeneSpring software. Clustering algorithm was based on standard correlation (r = 0.95). For hierarchical clustering, we included all genes with p < 0.05 after multiple testing correction. In order to determine the presence of significant linear relationships between gene expression and duration of untreated disease, linear regression was performed between the gene expression levels of each of the 79 genes and duration of untreated disease. All profiles are publicly accessible via NCBI GEO  (GSE11971).

### Quantitative Real Time-PCR verification (qRT-PCR)

Total cellular RNA was extracted using Trizol Reagent (Invitrogen Corp.) and subsequently DNase treated using DNA-free (Ambion, Austin, TX). Reverse transcription reactions were performed using Superscript III Reverse Transcriptase (Invitrogen Corp., Carlsbad, CA) and random hexamer primers. Relative cDNA quantification of smooth muscle myosin heavy chain (SMMHC), clusterin and an internal reference gene, β-actin, were done using a TaqMan PCR Core Reagent Kit (Applied Biosystems; Roche Molecular Systems, Inc., New Jersey) using fluorescence-based detection method (Applied Biosystems 7500 Fast Real-Time PCR System; Applied Biosystems, Foster City, CA). The PCR reaction was performed using standard methodology as previously described for each gene of interest and the β-actin reference gene was used to normalize input cDNA.

The primers and probe for SMMHC are as follows: 5'-CTGGGCAACGTAGTAAAACC-3' (Forward), 5'-TATAGCTCATTGCAGCCTCG-3' (Reverse), and 6FAM-ATAAGCTGGGCGTGGTGGTACACACCT-TAMRA (Probe) [50].

The primers and probe for Clusterin are as follows: 5'-GAGCAGCTGAACGAGCAGTTT-3' (Forward) 5'-CTTCGCCTTGCGTGAGGT-3' (Reverse) and 6FAM-ACTGGGTGTCCCGGCTGGCA-TAMRA (Probe) [51]

The primers and probe for β-actin are as follows: 5'-TGAGCGCGGCTACAGCTT-3' (Forward) 5'-TCCTTAATGTCACGCACGATTT-3' (Reverse) and 6FAM-ACCACCACGGCCGAGCGG-TAMRA (Probe) [52]

For relative cDNA quantification of class II antigen, HLA-DQA1 (#QT00060130), plexin D1 (#QT00036134), tenomodulin (#QT01024590), and an internal reference gene, β-actin (#QT00095431), QuantiTect Primer Assays and QuantiFast Syber Green PCR kits (Qiagen Inc., Valencia, CA) were used (Applied Biosystems 7500 Fast Real-Time PCR System; Applied Biosystems, Foster City, CA). The PCR reaction was performed according to manufacturer's protocol for these genes using β-actin reference gene to normalize input cDNA.

### Immunohistochemistry assay for mature dendritic cells

Three untreated girls with long duration of active JDM, were age-race matched with three girls with duration of untreated symptoms of less than 2 months and 3 age-matched healthy female muscle donors and studied for presence of mature dendritic cells. Serial 6 um-thick frozen muscle sections were fixed in cold anhydrous acetone. Sections were then blocked for 30 minutes in 10% normal goat or donkey sera and incubated with primary antibody overnight at 4°C. Monoclonal antibodies against DC-LAMP (Beckman coulter, CA), raised in mice, were used at a dilution of 1:10. Polyclonal antibody against BDCA2 (Santa Cruz Biotechnology, CA), raised in goats, were used at a dilution of 1:50. After 3 washes with 1× PBS, slides were incubated for 1 hour at room temperature with biotin conjugated secondary antibodies against mouse and goat respectively (Jackson Immunoresearch, PA). Subsequently, slides were stained with Vectastain Elite (Vector Laboratories, CA), followed by a BioGenex Liquid DAB Substrate Kit (yielding brown coloration at site of positive antibody binding). The slides were then counterstained with haematoxylin and Scott's bluing solution (Ricca Chemical, Texas), dehydrated, and prepared for viewing.

## Authors' contributions

Y-WC oversaw the expression profiling, qRT-PCR and IHC experiments, determined data analysis strategies and performed the analysis, interpreted the profiling, qRT-PCR and IHC data, and prepared the manuscript. RS performed expression profiling and immunohistochemistry. HG-D performed statistical analyses and interpreted results. SS performed the qRT-PCR, supervised the muscle biopsy selection for study and wrote some of the experimental methods. NG performed the immunohistochemistry. LMP created the IRB protocol, oversaw the study, recruited the patients and supervised the biopsy collection, qRT-PCR and immunohistochemistry assays and participated in the literature review and manuscript preparation. All authors have read and approved the final manuscript.

## Supplementary Material

Additional file 1 **Supplemental table 1**. Complete gene list of genes in cluster A-F. All genes are in order shown in figure 1. Click here for file
